# Improving Equity of Access for Women Admitted to a Psychiatric Mother & Baby Unit in Kent

**DOI:** 10.1192/bjo.2024.401

**Published:** 2024-08-01

**Authors:** Tasneem Saumtally, Bosky Nair, Chidi Nwosu, Serena Merchant, Stephanie Archer

**Affiliations:** 1 Maidstone & Tunbridge Wells NHS Trust, Maidstone, United Kingdom; 2 Kent & Medway NHS & Social Care Trust, Maidstone, United Kingdom; 3 Kent & Medway NHS & Social Care Trust, Dartford, United Kingdom

## Abstract

**Aims:**

Rosewood Mother and Baby Unit (MBU) aims to provide inpatient psychiatric care to women with severe mental illness in Kent, Surrey & Sussex (KSS) in UK. Data from admissions during 2022 demonstrated discernible inequalities in admissions. A quality improvement project was undertaken to improve equity of access for admission of women with severe mental illness to Rosewood MBU, specifically, those under 18 years old, black and minority ethnicities, and across counties in KSS. The aim was to improve quality of care and patient experience for vulnerable groups across all ethnicities, not limited to their location or age.

**Methods:**

Baseline data of MBU admissions in 2022 was collated, including demographics, age, origin of referrals, diagnosis, ethnicity, length of stay, parity, previous MBU admissions, safeguarding concerns.

The project group, inclusive of an expert by lived experience, presented the data at various network meetings and stakeholder events that helped to share information and gather experiences on barriers to referrals to Rosewood MBU, barriers for women of black and ethnic minority background accessing MBU, differences in service provisions for under-18-year-old women with perinatal mental illness in various counties.

Data for women discharged from Rosewood MBU in 2023 was collated and compared against the findings from the previous year.

**Results:**

In the first half of 2022, there were 20% more women admitted from Kent than Surrey and Sussex combined. This improved following interventions with a better spread of patients across counties in July–December 2023 and a 11% rise in admissions of women from Surrey and Sussex compared with Kent.

There was a greater number of ethnicities and a greater number of women from different ethnicities admitted to Rosewood MBU when comparing 2022 with 2023, and specifically across each of the 6-monthly periods. In January–June 2022, 3 women of non-White British ethnicity were admitted, compared with a 400% increase in July–December 2023 with 12 women. Overall there was 260% increase in admissions of women of Black, Asian, Mixed, White-Other ethnicity in 2023 compared with 2022. Also, in 2023, there were 2 referrals and 1 admission of a women under the age of 18, compared with no referrals in 2022.

**Conclusion:**

Overall, the project demonstrates the positive impact of streamlining referral pathway, fostering collaborative working and integrating expertise from diverse professionals including experts by experience that can reduce service inequalities and improve patient outcomes.

